# Drive, instinct, reflex—Applications to treatment of anxiety, depressive and addictive disorders

**DOI:** 10.3389/fpsyg.2022.870415

**Published:** 2022-09-26

**Authors:** Brian Johnson, David Brand, Edward Zimmerman, Michael Kirsch

**Affiliations:** ^1^Department of Psychiatry, State University of New York, Upstate Medical University, Syracuse, NY, United States; ^2^Department of Psychology, Adelphi University, Garden City, NY, United States; ^3^Institute of Physiological Chemistry, University Hospital Essen, Essen, Germany

**Keywords:** drive (instinct), instinct, neuropsychoanalysis, SEEKING (enthusiasm) system, pleasure (principle), anxiety disorders, depressive disorder, addiction

## Abstract

The neuropsychoanalytic approach solves important aspects of how to use our understanding of the brain to treat patients. We describe the neurobiology underlying motivation for healthy behaviors and psychopathology. We have updated Freud’s original concepts of drive and instinct using neuropsychoanalysis in a way that conserves his insights while adding information that is of use in clinical treatment. Drive (Trieb) is a pressure to act on an internal stimulus. It has a motivational energic source, an aim, an object, and is terminated by the satisfaction of a surge of serotonin. An instinct (Instinkt) is an inherited pattern of behavior that varies little from species to species. Drives are created by internal/ventral brain factors. Instincts require input from the outside that arrive through dorsal brain structures. In our model unpleasure is the experience of unsatisfied drives while pleasure if fueled by a propitious human environment. Motivational concepts can be used guide clinical work. Sometimes what had previously described psychoanalytically as, “Internal conflict,” can be characterized neurobiologically as conflicts between different motivational systems. These motivational systems inform treatment of anxiety and depression, addiction in general and specific problems of opioid use disorder. Our description of motivation in addictive illness shows that the term, “reward system,” is incorrect, eliminating a source of stigmatizing addiction by suggesting that it is hedonistic. Understanding that motivational systems that have both psychological and brain correlates can be a basis for treating various disorders. Over many papers the authors have described the biology of drives, instincts, unpleasure and pleasure. We will start with a summary of our work, then show its clinical application.

## The problem of drive and instinct

There has been a confusion in psychoanalysis about the use of the terms drive and instinct since its inception. This lack of clarity may not have to do with Freud. Solms (2018a) explained, “Trieb—a pressure that is relatively indeterminate both as regards the behavior it induces and as regards the satisfying object—differs quite clearly from theories of instinct…an inherited behavior pattern peculiar to an animal species, varying little from one member of the species to another and unfolding in accordance with a temporal scheme which is generally resistant to change and apparently geared to a purpose.”

Compton (1983) review of “instinctual drives” included these four categories of theorists.

1. Those who follow Freud’s formulation of the death instinct.
2. Those who consider that aggression and sexuality may be viewed in a parallel way.
3. Those who reject the concept of aggression as an instinctual drive, seeing aggression as a result of frustration a non-specific tension reduction.
4. Those who would prefer to dispense with drive models.

The pleasure-unpleasure principle has also been seen as an aspect of drive theory. Brenner (2008) suggested that the drive for pleasure could replace the concept of drive as an explanation for motivation. Don’t we all just want what is most pleasant in life?

The pleasure principle is not just “wanting what is pleasant” but is closely related to what Freud referred to as, “The constancy principle,” which states that the psyche evolves as an “organ” programmed to maintain a relatively constant low level of psychic tension. As a corollary, the pleasure principle states that in almost every case, lowering the level of tension in the psyche is experienced as pleasurable while elevating the level of tension is uncomfortable (Solms, 2019). These concepts of the constancy and pleasure principles underly drive theory since it is specifically internal sources of psychic tension (the tension created by unfulfilled needs) which activates us to seek ways to moderate that tension.

Our solution about how to understand motivation follows the dual aspect monism approach of neuropsychoanalysis (Solms, 2019). We are all born with innate needs. Development involves learning, imperfectly, how to get our needs met. As the “science of subjectivity,” or study of consciousness, we should be able to identify a confluence of what we observe in the clinical setting of close observations of humans correlated with our understanding of neuroscience. This might be described as using neuroscience as the basic science of psychoanalysis (Johnson and Mosri, 2016). This approach elides the hermeneutic problem of each psychoanalyst’s opinions about what they see as the only source of information, by leavening each observation, when possible, with a brain-based correlate.

## The five sources of motivation

The basis of the hierarchy below is not only human observations but importantly, based on Panksepp’s foundational work that animals have developed motivational systems that we share. He traced the pathways for his seven basic Command Systems labeled: SEEKING, RAGE, FEAR, PANIC, LUST, CARE, and PLAY, and gave evidence that these affective/emotional systems are developed evolutionarily and are shared in all mammals (Panksepp and Biven, 2012). Since each Command System uses characteristic brain areas^1^ it should be possible in principle to verify the action of Panksepp’s systems experimentally.

Revisions of Panksepp’s model involve Johnson’s work on cathexis (Johnson, 2008) and on drive reduction (Johnson, 2013), Kirsch’s work on the effect of hormones and serotonin on drive (Kirsch and Mertens, 2018; Kirsch, 2019) and more recent work on addiction (Ringwood et al., 2021). We will explain the motivational systems including what a drive is and how it is different from an instinct. The motivational systems are a neuropsychoanalytic model to be used for patient care. They are listed from #1, SEEKING to #5, pleasure, from the most to least powerful force.

### SEEKING

SEEKING starts at the *ventral tegmental area*, runs along the basal forebrain through the *lateral hypothalamus*, and has its first synapse at the *nucleus accumbens*. From there branches run through the *basal ganglia* that produce motor activity. It is the “goad without a goal,” as Panksepp termed it. It can be described as appetitive investigation of the environment. Us humans always want to find out what is around us.

SEEKING runs on dopamine. No matter what goes on with other neurotransmitters, dopamine always modulates the activity of SEEKING. When you are asleep you pursue what you want in dreams, Freud’s, “Every dream starts with a wish.” SEEKING is the most powerful of the neural systems and is also of key importance for the other Command Systems.

SEEKING can be taken over by addictive drugs. By various mechanisms (Johnson, 2009; Ringwood et al., 2021) addicted persons are mainly surveying their environment for drugs, urgently wanted, thereby outcompeting other motivators. Evidence for the power of addictive drugs in SEEKING is the prevalence of death from drug addiction; nearly 1/4 American deaths and a substantial cause of death all over the world (Johnson, 2018).

The SEEKING Command System is of key importance because Panksepp noted that SEEKING is “*the ‘granddaddy’ of all the emotional systems*” (Panksepp and Biven, 2012, p. 86). Thus, SEEKING is able to modulate all of the other Command Systems, probably by stimulating orexinergic neurons of the *lateral hypothalamus* (Kirsch, 2019).

### Drives

The *lateral hypothalamus* is perfectly positioned anatomically to tune the “goad without a goal” to a specific purpose. A hormone from the periphery lodges in the *lateral hypothalamus* directing SEEKING (Kirsch and Mertens, 2018; Kirsch, 2019). This is our unique definition of a drive. The drive terminates with a huge upsurge in serotonin (Kirsch and Mertens, 2018; Kirsch, 2019). Occupation of the receptor 5-HT_2C_ on tonically bfiring neurons leads to an inhibition of dopamine release (Kirsch and Buchholz, 2020). With the drive terminated, often another takes its place (Table 1—Drive, hormone).

**TABLE 1:** Somatic sources of drive motivation (Johnson, 2008; Kirsch and Mertens, 2018; Kirsch and Buchholz, 2020).

Drive	Hormone
Sleep	Adenosine
Sex	Estradiol/testosterone
Hunger	Ghrelin
Thirst	Angiotensin II
Relationships (cathexis)	Oxytocin (women and infants) Vasopressin (men)

The executing hormone of the sexual drive, i.e., estradiol/testosterone, modulates the activity of two of Panksepp’s affective systems (i.e., LUST and CARE) directly by addressing key brain areas of these affective systems and indirectly by addressing the SEEKING Command System (Kirsch, 2019). According to Panksepp, the activity of LUST and CARE depends additionally on oxytocin networking (Panksepp and Biven, 2012) (chapter 7 and chapter 8), i.e., on the executing hormone of attachment drives (Table 1). Since the activity of LUST and CARE is strongly under the control of executing drive hormones, these systems are separated from the other four Command Systems. The other four will be described as instinct later.

Panksepp and Biven (2012, p. 297) described LUST and CARE as having a *lateral hypothalamic* origin. Oxytocin is produced profusely during parturition to mark the child as an important object. Cathexis requires simultaneous engagement of dopamine, oxytocin and endogenous morphine (Johnson, 2008). Orgasm is an especially potent creator of cathexis because oxytocin and endogenous morphine synergistically reinforce each other (Johnson, 2008). Table 2 shows the relationship of drive, instinct and reflex. Table 3 shows their differences.

**TABLE 2:** Interrelationship of reflex, drive and instinct.

Construct	How it is communicated to/in the brain
Reflexes (many)	Activation of sensory nerves
Essential drives (5 total—Table 1)	Release of executing hormones lodging in the lateral hypothalamus
Instincts (4 total—section 4)	Activated by sensory nerves or reflexes Regulated by essential drives

**TABLE 3:** Principal differences between drives, reflexes and instincts.

Entity/*example*	Onset	Trigger communication	Typical involvement of brain areas
Drive/*hunger*	Organ excitation/*stomach*	(Peripheral) hormones/*ghrelin*	Lateral hypothalamus plus a drive specific brain area (Kirsch and Mertens, 2018) (i.e., the nucleus arcuatus (Panksepp and Biven, 2012) in the case of hunger)
Reflex/*acoustic startle reflex*	Excitation of sensory nerves/*excitation of acoustic neurons of the central nervous system*	Hormone free sensory nerve activation/*auditory nerve fibers in the ear synapses to the cochlear root neurons*	Autonomic reflex arc (spinal cord), somatic reflex arc (brainstem)/*cochlear root neurons synapse to the cells in the nucleus reticularis pontine caudalis* (Zhang et al., 2022)
Instinct/*fear*	External/internal threat A) Activation of the typical neural areas by drive hormones/*SEEKING, FEAR and RAGE* (Kirsch, 2019) B) Activation of the typical neural areas by a reflex/*the processing of the acoustic startle reflex activates the amygdala* (Lee and Davis, 1997; Pissiota et al., 2003; Milrod et al., 2007) C) Activation of instincts (esp. fear) by the autonomous nervous system in a reflex free manner?	Activation of sensory nerves by the autonomous nervous system when neither drive nor reflex operate	Brain areas of the corresponding Command System/*FEAR: central and lateral amygdala, medial hypothalamic to dorsal periaqueductal gray to nucleus reticularis pontine caudalis* (Watt, 2017)

#### How drives switch on and off

Switching of drives is apparent in human experience. For example, serotonin is zero during stage 4 and REM sleep (Saper et al., 2010). One wakes up with a surge of serotonin (Kirsch and Mertens, 2018), “Wow, what a wonderful day!” (Neurotransmitter regulation of sleep is complex. This simplification follows the cited references that serotonin terminates drive).

But then you notice the attractive partner next to you. Testosterone or more likely estradiol, produced directly in women as well as a metabolic product of testosterone for men, has lodged in your *lateral hypothalamus*. You may start with touch, activating the PLAY instinctual system. Things get more serious as LUST engages.

After your orgasm has produces a surge of serotonin, you think, “That was great, but what’s for breakfast?” because ghrelin from your stomach has lodged in your *lateral hypothalamus*. Angiotensin turns on thirst. Coffee hydrates, turning off angiotensin II, and turns off adenosine, making you even more awake.

At night the switching may be reversed. Orgasm switches off sex and the sleep drive is now apparent. The high level of adenosine needs to be turned back into cyclic AMP during sleep to fuel tomorrow’s activities.

What about relationships? If you are walking in a crowded city, it is annoying when strangers brush by you. The psychoanalytic term “cathexis” requires that oxytocin in the *lateral hypothalamus* marks someone as important to you. This phenomenon is intensified by orgasm with a person (Johnson, 2008). Now you really want to see that person again! The drive for sex and the drive for relatedness conflate making it complicated to start having sex with someone who is not right for you. We will see the conflict below described when we consider pleasure.

The oscillation of serotonin up and down that makes life exciting can be ruined by two common problems. The first is the use of addictive drugs. They take over the SEEKING system. Now the goal of your goad is obtaining drugs. Complicating this is that addictive drugs turn on serotonin (Ringwood et al., 2021). You lose the wonderful oscillation. You are a flat, unrelated person chasing drugs that make you miserable.

The other way to ruin the drive system is to prescribe serotoninergic antidepressants, SSRI and SNRI drugs. Not only do these drugs impair sexual functioning for most users, Clayton et al. (2006) they create a flat existence that patients struggle to describe. They will say things like, “My depression is better but I feel like I am observing my life through a pane of glass.” We speculate that these drugs create a high floor of serotonin tone, reducing the magnitude of fluctuation involved in drive function.

Drives turn on and off. The experience of having no drive turned on because one is well-slept, well fed and watered, satisfied sexually and has lots of close relationships allows SEEKING to be the preeminent experience. This pleasant state cannot go on long. Before one knows it the need for food, water, sex, closeness or sleep recurs.

### Unpleasure

This is Freud’s term. “Sensations of a pleasurable nature have not anything inherently impelling about them, whereas unpleasurable ones have it in the highest degree. The latter impel toward change, toward discharge, and that is why we interpret unpleasure as implying a heightening and pleasure a lowering of energetic cathexis…Let us call what becomes conscious as pleasure and unpleasure a quantitative and qualitative ‘something’…This ‘something’ behaves like a repressed impulse. It can exert driving force without the ego noticing the compulsion. Not until there is resistance to the compulsion, a hold-up in the discharge-reaction, does the ‘something’ at once become conscious as unpleasure” (Freud, 1966). Freud seemed to have recognized that unpleasure is not simply the opposite of pleasure. It is really something quite different and much more powerful. We reflect that in our understanding of its neurobiological source, the experience of urgently wanting to satisfy something that SEEKING is demanding.

If one would like to sleep but have to stay up with a sick child, if one wakes up next to an attractive partner but she/he doesn’t want to make love, if you would like to eat but you have to stay at work, and of course, if your brain is calling out for an addictive drug—it causes a feeling of misery. You hope your child goes to sleep so you can go to sleep, or that you can seduce your partner, or that you have good things in your refrigerator for after work, or that you can get to the cash machine, then find your drug dealer. Unpleasure can be lowered by pursuing your goal in a dream.

Unpleasure is more powerful as a driver of drug use than as a driver of ordinary drive goals. One will stay awake or not eat in order to consume cocaine (Johnson, 2009). Lying and using go together because the unpleasure of not having drugs is more important than relationships; one gives up close relationships for drugs. Addicted persons lie to their children and rob their grandmothers.

Unless one has powerful allies, drugs are lethal. The SEEKING system is the source of one’s will (Johnson, 2013). The innovation of Alcoholics Anonymous (step 3), “Made a decision to turn our will and our lives over to the care of God as we understood him,” is the way to get around this neurobiological reality. Addiction is the only disease where not asking for help is a central aspect of the illness. Although not conscious, the conflict includes that asking for help is linked for the actively addicted person with abstinence. One way to describe this is the AA aphorism, “It is hard to stop drinking when you are drinking.” Actively addicted persons know that asking for help and drinking are in conflict. Consciously focusing on relatedness by seeing a therapist, being honest, and perhaps also using 12 step allies, balances the unpleasure of tolerating abstinence despite drugs being urgently wanted against the (weaker—see below) pleasure of being with people.

### Instincts

How do we differentiate instincts from drives? As described by Panksepp (1998) and Panksepp and Biven (2012) instincts are shared by all mammalian animals. These are four of Panksepp’s seven systems; PANIC, FEAR, RAGE, PLAY. We are using one of Solms’ most important concepts (Solms, 2013). The ventral brain is turned inward to find out what the body needs; food, water, sleep, sex, companionship. The dorsal brain is turned outward to understand the environment, including the state of the peripheral body such as pain. When the SEEKING system is switched on to one of these Command Systems the instinct dominates thinking and behavior.

Information collected by *dorsal brain areas* such as the *insula* are required to turn on instinctual behaviors. Instincts originate with outside stimuli that tune SEEKING to behaviors that are required for survival. They are switched on by environmental factors. PANIC, FEAR and RAGE are turned on indirectly via orexin release during SEEKING activities (Kirsch, 2019).

The PANIC system turns on separation distress. PANIC is interpersonal. We routinely say to patients with panic attacks that they are about being alone, even if there are people all around. The people in one’s family need to be warm and loving for PANIC to be turned off. Cold, uncaring relationships trigger PANIC (Panksepp, 1998; Panksepp and Biven, 2012). The PANIC system is anatomically directly on top of SEEKING (Coenen et al., 2012) as if it is there to shut off SEEKING to produce “neurovegetative” symptoms of depression. One can’t sleep, eat, one does not want sex. One can’t even pay attention when SEEKING is down-regulated by PANIC.

FEAR is tissue-protective (Panksepp and Biven, 2012). FEAR can be caused by persons who are menacing but also by non-human dangers such as a predatory animal, or by walking to the edge of a cliff. FEAR is not necessarily interpersonal. It has to do with danger in the environment. FEAR is turned off when tissue damage has been avoided.

RAGE is turned on by unpleasant impingement (Panksepp and Biven, 2012, p.149). RAGE is also turned on when anticipated rewards are denied by another (Panksepp and Biven, 2012, p. 149). We hypothesize that RAGE is turned off by termination of the offending person’s behavior; either an apology or a retreat. We would suggest that unpleasant as it is, the RAGE circuit helps cultivate good relationships. Well-related people get angry easily and immediately deal with the source of impingement.

PLAY is turned on by having a potential play partner in the vicinity. Touch mediated by thalamic areas is an important basis of PLAY (Panksepp and Biven, 2012). Young animals, whether rat or human, want to wrestle. There seems to be a limit to how much one wants to play. It is terminated by satisfaction.

Drives are internal and instincts have to do with interactions in the environment.^2^ One can eat or sleep by oneself, or use drugs alone.

The term, “Partying,” is part of the denial system of drug addiction, as if drug use is a social activity. There is nothing interpersonal about addiction as a neurobiological entity. If someone dies at the crack house sometimes the body is put out in the hallway and drug use continues. Nothing playful about that! Cocaine has taken the Command System SEEKING, rendering drives less relevant to life (Ringwood et al., 2021). The behavior is purely internally driven. PANIC, RAGE, FEAR and PLAY are turned off.

### Pleasure

Oddly, pleasure is the weakest determinant of human activities. If one inhales a cigarette, one will urgently want to do it again soon. Typically for an addictive illness, the unpleasure of needing the next cigarette is a potent driver of behavior. But if you eat dinner at a great restaurant, you have no urge to go back for breakfast. Human companionship is the main component of pleasure (Panksepp et al., 1980). How much more pleasant to have dinner with a friend than alone!

The weakness of pleasure results in complaints about function that bring patients to treatment. “I keep dating the same woman/man all the time. I fall in love and then am miserable.” Cathexis is built on previous relationships. If one’s parents were unpleasant, one will be attracted to unpleasant people, then complain about how unpleasant it is to be with them. We all assume that we want what we like, but wanting has to do with SEEKING (Wright and Panksepp, 2011). Pleasure is not a brain pathway but rather a distributed state that has much to do with endogenous opioids (Berridge and Kringelbach, 2015).

Opioid maintenance makes human interactions aversive. Autism featuring gaze avoidance, reluctance to speak and repetitive behaviors that ward off human interactions may be due to high levels of endogenous opioid make human interactions aversive (Anugu et al., 2021). Opioid maintenance therapy makes people behave as if they are autistic. Is it worth taking exogenous opioid maintenance to lower the risk of death from using illicit opioids? Practice right now is to make this decision for all patients in the affirmative rather than allow patients to choose.

We have represented opioid tone in the central nervous system with a quadratic equation:


Pleasure⁢(x)=4-(x-3)

x=opioid⁢tone,limit⁢x=0<x<6

**FIGURE 1:**
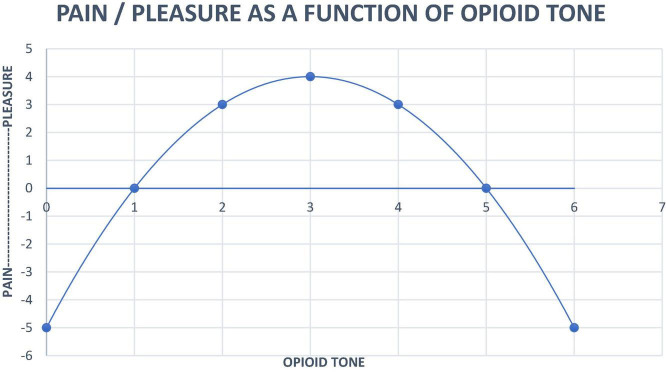
Neurobiological systems engineering model of the relationship of pain, pleasure and opioid tone.

This Equation models our clinical observations as follows:

• Healthy persons use friendly contact to increase opioid tone and solitude to reduce opioid tone, keeping it between 2 and 4 (numbers refer to the x-axis of Figure 1).

• When healthy persons feel the distress of loneliness they seek comfort through the proximity of others to increase opioid tone. It feels good.
• Prolonged intense contact causes discomfort. Healthy humans seek solitude to reduce opioid tone to a pleasant level.
• Healthy persons can engage and disengage flexibly.
• Psychopathology such as PTSD, ADHD or borderline personality organization force unrelatedness that results in opioid tone below 1 (x-axis). Such isolated individuals are prone to opioid addiction (Johnson and Faraone, 2013). Opioids might be characterized as, “A person in a pill.”
• Opioid detoxification results in opioid tone below 1, militating toward relapse to opioid use. This state can be ameliorated with low dose naltrexone (Jackson et al., 2021).
• Fibromyalgia symptoms are consistent with an autoimmune disease that strikes mu opioid receptors, also setting opioid tone below 1 (Jackson et al., 2021).
• Autism symptoms such as gaze avoidance, lack of language and social interaction suggest opioid tone past 5 where augmentation of opioid tone is painful. High dose naltrexone can be used to move tone back toward the midline zone (2– 4 on the x-axis) (Anugu et al., 2021).
• Opioid maintenance also moves opioid tone past 5, resulting in avoidance of human interactions. Using this idea to create a contingency management “reward” of lowering psychotherapy frequency (emotional contact) for negative urine drug screens during buprenorphine maintenance of pregnant women resulted in a high quit rate for addictive drugs and no clinically significant neonatal abstinence syndrome (Tabi et al., 2020).

### Complications and nuances

Dreams are not simply guarding sleep. Every drive is connected with the FEAR system (Kirsch, 2019; Kirsch and Buchholz, 2020). PTSD dreams may start with a wish, but may also start with a fear that is marked by SEEKING. If a person has been nearly killed, the danger may be encountered by dreams, waking the person in fear as if to deal with the threat. If the human environment lacks contact, PANIC may be encountered in dreaming—being alone. RAGE competes with the drive for sleep, rendering sleep impossible. In contrast, PLAY and SEEKING share overlapping pathways, allowing play partners to appear in the dream, protecting sleep.

Fantasies may inadequately produce pleasure and drive reduction. The concept that drive reduction requires sequential engagement of SEEKING, then consummatory pleasure (Johnson, 2013), explains why real sex partners provide more than fantasy partners experienced as part of masturbation. Fantasies may be conscious, allowing planning, or unconscious, provoking defenses that determine behaviors that can’t be modified until enacted with a therapist who can help the patient put the unconscious fantasy into manipulable words.

Behaviors are action plans that are procedural not explicit (Solms, 2018b). The process of interpretation (Kernberg, 2016) involves putting implicit behaviors into words via clarification, confrontation, defense and transference interpretation. The procedural behaviors are not extinguished, but rather compete against conscious overriding of previously inchoate urges. Neurotic and addictive behaviors can be modified by the combination of interpersonal support and more conscious behavior.

The hormones of the Freudian drives have the capability to turn on FEAR whereas the mother-infant tie (and presumably also the ones of the other attachment drives) have the capability to turn off FEAR. Thus, drives can act as regulators of FEAR.

#### Reflex

An instinct can also be activated by a reflex. When we accidently put our hand on a hot stove, FEAR is activated during the withdrawal of our hand. The next time we see the stove, FEAR is turned on.

## Applications to psychiatric practice

There are profound implications of these behavioral motivators. These will be listed by headings.

### The psychoanalytic concept of conflict

While these systems are not the only source of conflict, this system of understanding motivation clarifies some conceptualizations/interpretations of conflicts. For example, McWilliams (McWilliams, 2004; Watt and Panksepp, 2009) described a woman with a brutal father who consciously sought to not recreate the unpleasantness of her relationship with her father by choosing a pacifist husband. Unfortunately, it became apparent to the patient that pacifism was an attempt in her husband to undo his sadism. The paternal cathexis (drive), a more powerful a motivator of behavior than pleasure, had determined her choice. Dr. McWilliams goal as the psychoanalyst was to help her patient be conscious of this more powerful motivator that had rendered her choice unpleasant.

More broadly, while we all assume we want what we like, conflicts in motivational systems are built into the brain. Cathexis is the tuning of the SEEKING system to the kinds of persons one grew up with. If they were pleasant and loving, there is likely to be a weak cathexis for these qualities in adult relationships. If they were aversive, abusive, abandoning and/or neglectful there is likely to be a strong cathexis, built on early relationships with closeness augmented by FEAR, PANIC, and RAGE, for this kind of adult relationship. Patients like Dr. McWilliams’ arrive for treatment complaining that their relationships are unpleasant despite their best efforts.

The type of relationship that is unpleasant may be recreated with the therapist. This phenomenon drove Freud in “Beyond the pleasure principle” to posit a death instinct. But what lies beyond the pleasure principle, as a more potent motivator of relational behavior, is drive/cathexis (Johnson, 2008).

What Freud meant by the death instinct was a sort of surrender to oblivion—a retreat from the world, from SEEKING anything at all, to stop trying. The state that opioid addicted persons are trying to achieve is just this sort of oblivion. They cannot find satisfaction in their engagement with the world and want to flee from it into a cocoon barely living state. For Freud, what was beyond the pleasure principle was the abandonment of the pursuit of the kinds of things that our “life” drives push us toward in the world of others. The opioid coma that addicted persons seek comes close to that.

### One vs. two person psychoanalytic theory

We can see that this is a false dichotomy. The human infant is born with innate needs; drives. The storm of oxytocin at birth creates in both individuals a strong cathexis, the mother-infant bond (Kirsch and Buchholz, 2020). Eventually the infant builds up an oxytocin—dependent cathexis with all other main caregivers.

Development is the process of exploring and imperfectly solving how to meet our needs (Tabi et al., 2020). Humans are social animals. We SEEK engagement. Our interpersonal experience is shaped by drive and instinct, unpleasure and pleasure. Entering into an intense therapeutic relationship with any patient creates a two—person field of previous cathected relationships for both parties; the transference and the countertransference. How to negotiate one’s countertransference while interpreting the transference with the humble and helpless attitude that one is only partly conscious about the interaction is essential to good technique.

### Treating “anxiety and depression”

As weak a motivator pleasure is, opioid tone at the top of the inverse U function makes one calm and happy. If one has been able to construct a human environment full of well-intentioned people, one might never need to see a psychotherapist. If one is bad at relationships it is difficult to regulate opioid tone. One slides down the left side of the inverse U. “Anxiety” is a signal from the PANIC system that human contact is needed. Patients with panic attacks can have their difficulties with relatedness addressed with psychoanalytic therapy (Modell et al., 1997).

Think of McWilliams’ patient. She is married but feels alone. Anxiety is the signal that something is wrong but the signal is difficult to decode. Perhaps she will set up a relationship with Dr. McWilliams that is also distant. Dr. McWilliams will become aware of this by examining a countertransference that is unique to this patient.

If the distance is marked and enduring, depression ensues (Johnson, 1992). This sets up a classic problem, the question of medications. Some practitioners pride themselves on symptom reduction and provide benzodiazepines. This just builds in a chemical distance without solving the problems. Some practitioners have been taught that SSRIs are the first line medications for anxiety and depression. This builds in another kind of distance by raising baseline serotonin tone and reducing the fluctuations in serotonin that make life intensely pleasant. For Dr. McWilliams’ patient it might create a secondary problem that not only is she turned off sexually by her husband’s sadism, she is also turned off chemically by a SSRI medication that reduces sexual interest, lubrication and orgasm (Johnson, 1992).

Feelings mean something. The effect size for antidepressants is about 0.3 and for psychoanalytic therapy starts at 0.8 and moves upward in different studies (Tabi et al., 2020). Why psychotherapy is not widely recommended to treat anxiety and depression becomes a reasonable question. The goal of examining the transference relationship to minimize interpersonal distance in general, if realized sufficiently, restores opioid tone to the top of the inverse U. The patient leaves therapy feeling better.

### Treatment of addiction

In 1789 tobacco and alcohol were legal addictive drugs in the United States. Marijuana is being added to the list. Other addictive drugs afflict a much smaller percentage in the population. Drug use is characteristically adopted during teenage years to shut off PANIC, RAGE, and FEAR (Ringwood et al., 2021).

The imbrication of drugs into the SEEKING system creates a permanent change in function. Actively addicted patients complain that they urgently want the drug even while suffering unpleasant consequences from use. There is nothing nice about having an addiction.

Seeing the conflict between urgent drug SEEKING to ward off unpleasure, and the pleasure of being warmly related, gives the psychotherapist purchase to use clarification and confrontation about drug use to bring to consciousness this conflict. The model that drug addiction takes over SEEKING to render it insensitive to the drive to be related helps understand why the illness that routinely results in not wanting treatment; exactly because the addicted person has given up relatedness for drugs. When the therapist successfully engages with a newly sober patient the hostility of drug use may enter the transference relationship along with elements of childhood trauma (Panksepp, 1981; Johnson, 2010).

Opioid Use Disorder is subject to the inverse U function shown in Figure 1. Opioid maintenance makes patients untreatable by psychotherapy because endogenous opioid tone is the driver of relatedness (Carroll and Weiss, 2017). Emotional closeness hurts when opioid tone is high! This is reflected in the broader psychotherapy literature that interventions that require a therapeutic alliance have no impact during buprenorphine maintenance (Johnson et al., 2014).

The inverse U function explains the vulnerability of persons with personality disorders to opioid use disorder. Their difficulty with relationships creates enduring low opioid tone that can be corrected with a “person in a pill.” Opioids feel like people. Opponent process ensues making the distance/depression worse. Every dose of opioid feels good even as the tone created by the impact of exogenous opioids decreases the efficiency of the corresponding receptor system thereby enhancing drivers of misery; anxiety, depression and pain. This phenomenon probably results in the problem of “isolation” as a core aspect of opioid addiction.

Our formulation about addiction calls into question the term, “reward system,” that is ubiquitous in neuroscience articles. Behavioral psychology specifically disregarded anything about the brain (Panksepp, 1998, p. 12). The term “reward” was used as anything that caused animals to enact a specific behavior more frequently, unconnected with any conceptual thinking about the brain.

Our hierarchy of motivators suggests that by taking over SEEKING, and producing frequent experiences of awful unpleasure because drugs are not immediately available, pleasure is negated by drug addiction. Using the term “reward” for the effects of addictive drug use creates stigma, as if use of drugs was hedonistic rather than a desperate attempt to avoid enduring PANIC, FEAR, and RAGE caused by a disadvantageous human environment. Drugs shut off feelings. Craving/SEEKING drugs creates unpleasure and the misery of constantly high serotonin tone, making a bad situation worse.

SEEKING that is not corrupted by addictive drug use is a pleasant expectancy that good things may happen (Panksepp and Biven, 2012). Learning takes place in the context of both positive and negative events (Panksepp and Biven, 2012, p. 105–106). We learn how to increase good outcomes and minimize bad outcomes.

## Conclusion

“Science is not really about ‘right,’, ‘wrong,’ ‘true,’ or ‘false.’ Theories should be evaluated as more or less ‘useful within a certain context” (Davies, 2021). Our approach is the product of reverberation between clinical observation and brain science. Having these dual aspects of information allows for a more robust grounding of both theory and treatment (Govrin, 2006).

We have updated Freud’s original concepts of drive and instinct in a way that conserves his original insights. Drive is a pressure to act on an internal stimulus. It has a motivational energic source, an aim, an object, and is terminated by the satisfaction of a surge of serotonin. Drive reduction requires SEEKING/exploration followed by satisfaction/consummatory outcomes. An instinct is an inherited pattern of behavior that varies little from species to species. Drives are created by internal/ventral brain areas and instincts require input from the outside that come in from dorsal structures. Instincts can be turned on by drives but drives cannot be turned on by instincts. For example, the drive for sex can turn on FEAR. Hunger can turn on RAGE. But the PLAY, FEAR, RAGE and PANIC systems do not turn on any drives because they are unable to induce the release of hormones such as estradiol/testosterone or ghrelin.

In terms of Compton’s four camps of psychoanalytic theorizing on drive and instinct:

1. From current insights, the death instinct (Freud, 1920) is rendered untenable. What lies beyond pleasure are the four more powerful motivational factors; SEEKING, drive, unpleasure and instinct.
2. Aggression and sexuality are not exactly parallel phenomena. Aggression might best be viewed as SEEKING. We SEEK sex because estradiol/testosterone has tuned SEEKING to sexuality. Other drives can be aggressively sought. The wolf chases the rabbit because of ghrelin/SEEKING.
3. Aggression as a result of frustration, a non-specific tension, is covered by our use of (and Freud’s use of) the term unpleasure. Tension reduction is a property of termination of drive by a surge of serotonin. RAGE is an instinct that has to do with impingement from without. The unpleasure of unmet drives and RAGE are two different sources of frustration.
4. We are we all born in an interpersonal matrix. Development has to do with learning how to have our needs met (Solms, 2018c) within that matrix. Humans are social animals. There is no conflict between drive and unique experiences, including in the psychotherapy dyad. Relational psychoanalysis can coexist with drive theory.

Reflexes are noted to be a separate phenomenon. We all have reflexes to defecate when the colon is full at birth. Around age 2 this reflex can be modified by learning in an interpersonal network of caregivers.

At the same time as resolving a problem in psychoanalytic theory this way of conceptualizing drive and instinct helps create new aspects of psychiatric treatment. Specific disorders: anxiety disorders, depressive disorders, addictions in general, and opioid addiction in particular are all amenable to improved care because of the update of psychoanalytic theory.

The hierarchy of motivational systems: SEEKING, drive, unpleasure, instinct, and pleasure, move psychoanalysis into the scientific mainstream. They have an impact on mental health treatment in general. We have solved one aspect of the problem of how to work neurobiology into general psychiatric practice.

## Data availability statement

The original contributions presented in this study are included in the article/supplementary material, further inquiries can be directed to the corresponding author/s.

## Author contributions

BJ wrote the first draft. MK’s concepts were of the central importance. All authors participated in contributing important concepts.

## Footnotes

1 The classification of Command Systems was evaluated according to Watt (2017). The following major structures were given as constituents of the seven Command Systems. SEEKING: *ventral tegmental area, lateral hypothalamus, periaqueductal gray, nucleus accumbens*. RAGE: *medial amygdala, bed nucleus of stria terminalis, anterior and ventromedial and perifornical hypothalamus, dorsal periaqueductal gray*. FEAR: *central and lateral amygdala, medial hypothalamic to dorsal periaqueductal gray to nucleus reticularis pontine caudalis*. LUST: *bed nucleus of stria terminalis, corticomedial amygdala to preoptic and ventromedial hypothalamus to lateral ventral periaqueductal gray*. CARE: *anterior cingulate to bed nucleus of stria terminalis to preoptic hypothalamic to ventral tegmental area to more ventral periaqueductal gray*. PANIC: *anterior cingulate, anterior thalamus to bed nucleus of stria terminalis, ventral septum to midline and dorsomedial thalamus to dorsal preoptic hypothalamus to more dorsal periaqueductal gray*. PLAY: *parafascicular/centromedian thalamus, dorsomedial thalamus, posterior thalamus, projecting to ventral periaqueductal gray* (septum inhibitors).

2 Nevertheless, a Freudian drive can (but do not necessarily have to) activate FEAR and RAGE (Kirsch, 2019) whereas an instinct is unable to turn on a drive.
